# *meso*-(1S*,21R*)-25-Methyl-8,11,14-trioxa-22,24,25-triaza­tetra­cyclo­[19.3.1.0^2,7^.0^15,20^]penta­cosa-2,4,6,15(20),16,18-hexa­ene-23-thione chloro­form monosolvate

**DOI:** 10.1107/S1600536812037051

**Published:** 2012-09-05

**Authors:** Truong Hong Hieu, Le Tuan Anh, Anatoly T. Soldatenkov, Vladimir V. Kurilkin, Victor N. Khrustalev

**Affiliations:** a Department of Chemistry, Vietnam National University, 144 Xuan Thuy, Cau Giay, Hanoi, Vietnam; b Organic Chemistry Department, Russian Peoples Friendship University, Miklukho-Maklaya St. 6, Moscow, 117198, Russia; c X-Ray Structural Centre, A.N. Nesmeyanov Institute of Organoelement Compounds, Russian Academy of Sciences, 28 Vavilov St., B-334, Moscow 119991, Russian Federation

## Abstract

The title compound crystallizes as a chloro­form solvate, C_20_H_23_N_3_O_3_S·CHCl_3_, with two crystallographically independent units. The independent units have distinctly different inter­action patterns between the aza­crown macrocycle and the chloro­form solvent mol­ecule. In one of them, the chloro­form mol­ecule forms C—H⋯N and Cl⋯H—C hydrogen bonds with the aza­crown macrocycle (as a proton donor and an acceptor, respectively), whereas in the other, one of the chloro­form mol­ecules is bound to the aza­crown macrocycle by an attractive Cl⋯O [3.080 (3) Å] inter­action. The aza­crown macrocycles of different units are structurally similar; the aza-14-crown-3-ether ring adopts a bowl conformation with dihedral angles between the planes of the fused benzene rings of 60.7 (1) and 68.0 (1)°. The triazinane­thione ring in both cases has a sofa conformation. The crystal packing is characterized by N—H⋯S, N—H⋯O, C—H⋯Cl and C—H⋯S hydrogen bonds.

## Related literature

For general background, see: Hiraoka (1982 ▶); Pedersen (1988 ▶); Gokel & Murillo (1996 ▶); Bradshaw & Izatt (1997 ▶). For related compounds, see: Levov *et al.* (2006 ▶, 2008 ▶); Anh *et al.* (2008 ▶, 2012*a* ▶,*b* ▶); Hieu *et al.* (2009 ▶, 2011 ▶); Khieu *et al.* (2011 ▶).
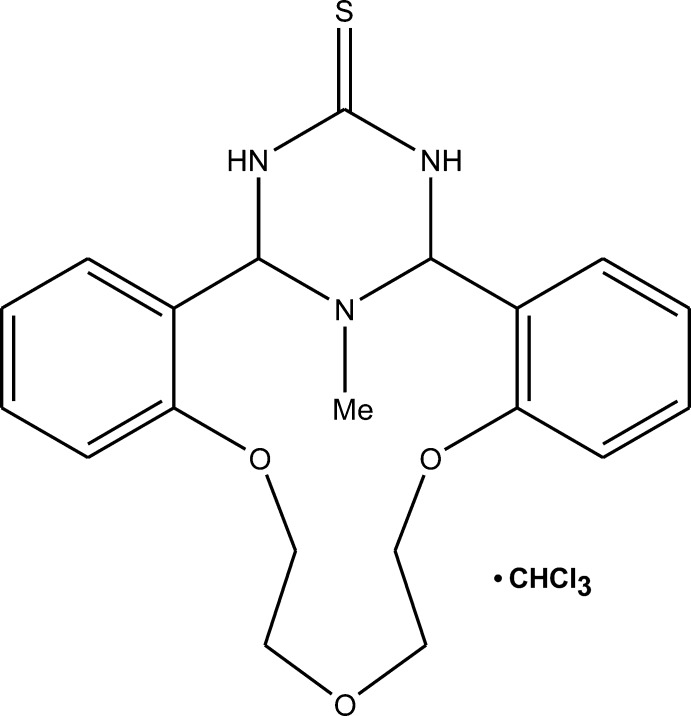


## Experimental

### 

#### Crystal data

C_20_H_23_N_3_O_3_S·CHCl_3_
*M* *_r_* = 504.84
Monoclinic, 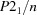

*a* = 17.8370 (5) Å
*b* = 13.9173 (4) Å
*c* = 19.0561 (6) Å
β = 99.222 (1)°
*V* = 4669.4 (2) Å^3^
*Z* = 8
Mo *K*α radiation
μ = 0.51 mm^−1^
*T* = 100 K
0.30 × 0.25 × 0.20 mm

#### Data collection

Bruker APEXII CCD diffractometer
Absorption correction: multi-scan (*SADABS*; Sheldrick, 2003 ▶) *T*_min_ = 0.862, *T*_max_ = 0.905
52534 measured reflections
11281 independent reflections
8711 reflections with *I* > 2σ(*I*)
*R*_int_ = 0.052

#### Refinement

*R*[*F* ^2^ > 2σ(*F* ^2^)] = 0.051
*wR*(*F* ^2^) = 0.145
*S* = 1.00
11281 reflections
561 parameters
H-atom parameters constrained
Δρ_max_ = 1.38 e Å^−3^
Δρ_min_ = −1.05 e Å^−3^

### 

Data collection: *APEX2* (Bruker, 2005 ▶); cell refinement: *SAINT* (Bruker, 2001 ▶); data reduction: *SAINT*; program(s) used to solve structure: *SHELXTL* (Sheldrick, 2008 ▶); program(s) used to refine structure: *SHELXTL*; molecular graphics: *SHELXTL*; software used to prepare material for publication: *SHELXTL*.

## Supplementary Material

Crystal structure: contains datablock(s) global, I. DOI: 10.1107/S1600536812037051/ld2071sup1.cif

Structure factors: contains datablock(s) I. DOI: 10.1107/S1600536812037051/ld2071Isup2.hkl

Supplementary material file. DOI: 10.1107/S1600536812037051/ld2071Isup3.cml

Additional supplementary materials:  crystallographic information; 3D view; checkCIF report

## Figures and Tables

**Table 1:** Hydrogen-bond geometry (Å, °)

*D*—H⋯*A*	*D*—H	H⋯*A*	*D*⋯*A*	*D*—H⋯*A*
N22—H22*N*⋯O11^i^	0.90	2.32	3.183 (2)	161
N24—H24*N*⋯S1^ii^	0.90	2.55	3.445 (2)	173
N48—H48*N*⋯O37^iii^	0.90	2.38	3.273 (3)	172
N50—H50*N*⋯S2^iv^	0.90	2.55	3.445 (2)	172
C10—H10*B*⋯S2^iv^	0.99	2.80	3.747 (2)	160
C21—H21⋯Cl3^i^	1.00	2.66	3.395 (2)	130
C26—H26*A*⋯Cl2	0.98	2.78	3.514 (2)	133
C36—H36*A*⋯S1^ii^	0.99	2.78	3.729 (3)	160
C43—H43⋯Cl3^v^	0.95	2.83	3.690 (3)	151
C53—H53⋯N25	1.00	2.46	3.353 (3)	149

Symmetry codes: (i) 
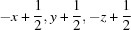
; (ii) 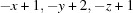; (iii) 
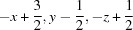
; (iv) 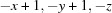; (v) 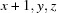.

**Fig. 1.**
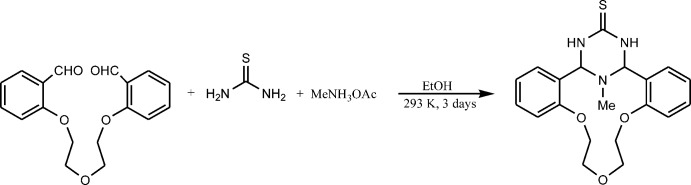
Multicomponent condensation of thiourea with 1,5-bis(2-formylphenoxy)-3-oxapentane and methylammonium acetate.

**Fig. 2.**
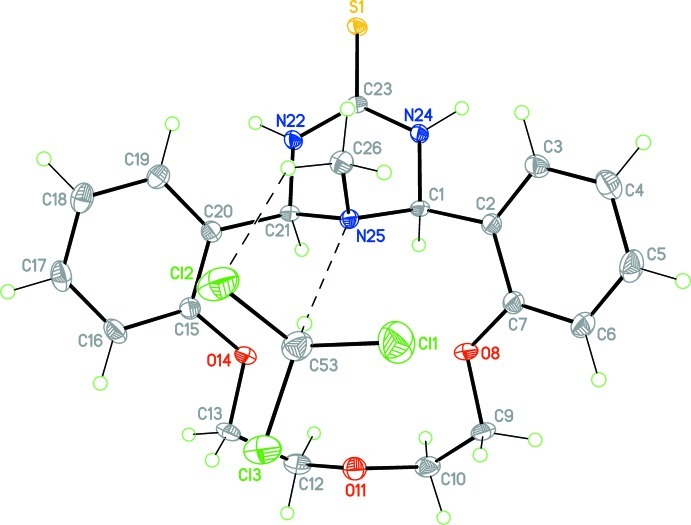
Molecular structure of I (first crystallographically independent I.CHCl3 unit is depicted). Displacement ellipsoids are shown at 50% probability level. Dashed lines indicate intermolecular hydrogen bonds and attractive O···Cl interaction. H atoms are presented as small spheres of arbitrary radius.

**Fig. 3.**
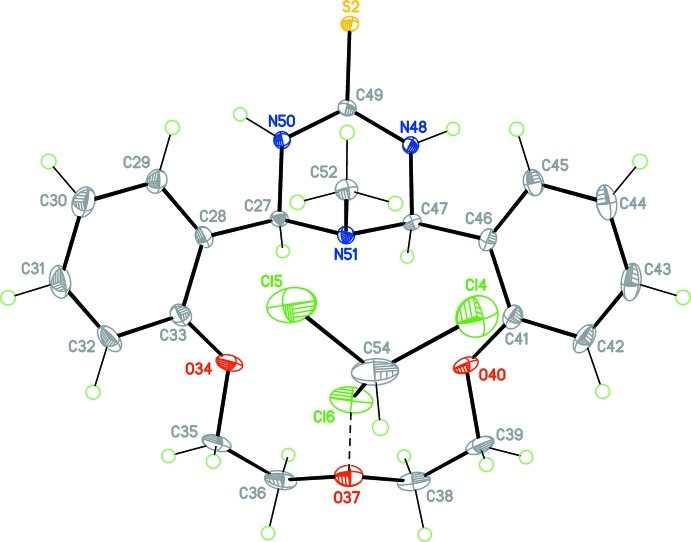
Molecular structure of I (second crystallographically independent I.CHCl3 unit is depicted). Displacement ellipsoids are shown at 50% probability level. Dashed lines indicate intermolecular hydrogen bonds and attractive O···Cl interaction. H atoms are presented as small spheres of arbitrary radius.

## supplementary crystallographic information

### Comment

Supramolecular chemistry of azacrown ethers draws a great attention of researchers during the last decades (Hiraoka, 1982; Pedersen, 1988; Gokel & Murillo, 1996; Bradshaw & Izatt, 1997). Recently, we have developed effective methods of synthesis of azacrown ethers containing piperidine (Levov *et al.*, 2006, 2008; Anh *et al.*, 2008, 2012*a*, 2012*b*), perhydropyrimidine (Hieu *et al.*, 2011) and perhydrotriazine (Hieu *et al.*, 2009; Khieu *et al.*, 2011) subunits.

In an attempt to apply these for a synthesis of a macrocyclic ligand with an *N*-methylsubstituted perhydrotriazine moiety, we studied the multicomponent condensation of thiourea with 1,5-bis(2-formylphenoxy)-3-oxapentane and methylammonium acetate. The reaction has proceeded smoothly under mild conditions to give the expected azacrown moiety in a good yield (Figure 1).

Compound I crystallizes as a chloroform solvate, *i. e.*, C_20_H_23_N_3_O_3_S.CHCl_3_, with two crystallographically independent units within the unit cell. These crystallographically independent units represent two different molecular I.CHCl_3_ associates distinguished by different interactions between I and CHCl_3_ counterparts. In one of the two associates, the chloroform molecule forms the two C53—H53···N25 (as a protonodonor) and Cl2···H26A—C26 (as a protonoacceptor) hydrogen bonds (Table 1, Figure 2a), whereas, in the other associate, the chloroform molecule is bound to the molecule I by the attractive Cl6···O37 (3.080 (3) Å) interaction (Figure 2b). The azacrown macrocycles of the different I.CHCl_3_ associates are structurally similar.

The aza-14-crown-3-ether ring adopts a bowl conformation. The configuration of the C7—O8—C9—C10 —O11—C12—C13—O14—C15 polyether chain is t–g(-)–t–t–g(+)–t (t = *trans*, 180°; g = *gauche*, ±60°). The dihedral angles between the planes of the benzene rings fused to the aza-14-crown-3-ether moiety are 60.69 (8) and 68.01 (5)° for two crystallographically independent molecules, respectively. The triazinanethione ring has a sofa conformation - the nitrogen atoms N22, N24, N48 and N50 have a trigonal-planar geometry (sums of the bond angles are 358.8, 360.0, 359.0 and 359.9°, respectively), while the nitrogen N25 and N51 atoms adopt a trigonal-pyramidal geometry (sums of the bond angles are 331.9 and 333.7°, respectively).

The molecule of I possesses two asymmetric centers at the C1 and C21 carbon atoms and represents a *meso*-form (an internal racemate).

In the crystal, the molecular I.CHCl_3_ associates are linked by the intermolecular N—H···S, N—H···O, C—H···Cl and C—H···S hydrogen bonds into a three-dimensional framework (Table 1).

### Experimental

Methylamine ammonium acetate (4.0 g, 44 mmol) was added to a solution of 1,5-bis(2-formylphenoxy)-3-oxapentane (1.57 g, 5.0 mmol) and thiourea (0.38 g, 5.0 mmol) in a mixture of ethanol (30 ml) and acetic acid (1 ml). The reaction mixture was stirred at 293 K for 3 days. At the end of the reaction, the formed precipitate was filtered off, washed with ethanol and re-crystallized from ethanol and ethylacetate (4:1) to give 1.19 g of white crystals of I. Yield is 61.8%. *M*.p. = 417–419 K. IR (KBr), ν/cm^-1^: 1603, 3215, 3332. ^1^HNMR (DMSO-*d*_6_, 400 MHz, 300 K): δ = 1.53 (s, 3H, CH_3_), 3.63 and 3.92 (both m, 3H and 5H, respectively, OCH_2_CH_2_OCH_2_CH_2_O), 6.21 (s, 2H, H1 and H21), 6.87 (d, 2H, *J* = 8.0, H6 and H16), 6.91 (tt, 2H, *J* = 7.6 and 0.8, H4 and H18), 7.25–7.30 (m, 4H, H_arom_), 8.27 (s, 2H, NH). Anal. Calcd for C_20_H_23_N_3_O_3_S: C, 62.32; H, 6.01; N, 10.90. Found: C, 62.51; H, 6.15; N, 10.86.

### Refinement

There are two relatively high positive peaks of 1.38 and 1.24 e Å^-3^ near the Cl5 and Cl4 chlorine atoms of the solvate chloroform molecule that indicate a slight disorder of the solvate molecule. However, due to the low contribution of the second component it was neglected.

The hydrogen atoms of the amino groups were localized in the difference-Fourier map and included in the refinement with fixed positional and isotropic displacement parameters [*U*ĩso~(H) = 1.2*U*~eq~(N)]. Other hydrogen atoms were placed in calculated positions with C—H = 0.95–1.00 Å and refined in the riding model with fixed isotropic displacement parameters [*U*ĩso~(H) = 1.5*U*~eq~(C) for the methyl group and 1.2*U*~eq~(C) for the other groups].

### Crystal data

**Table:**

C_20_H_23_N_3_O_3_S·CHCl_3_	*F*(000) = 2096
*M**_r_* = 504.84	*D*_x_ = 1.436 Mg m^−^^3^
Monoclinic, *P*2_1_/*n*	Mo *K*α radiation, λ = 0.71073 Å
Hall symbol: -P 2yn	Cell parameters from 8820 reflections
*a* = 17.8370 (5) Å	θ = 2.3–31.3°
*b* = 13.9173 (4) Å	µ = 0.51 mm^−^^1^
*c* = 19.0561 (6) Å	*T* = 100 K
β = 99.222 (1)°	Prism, colourless
*V* = 4669.4 (2) Å^3^	0.30 × 0.25 × 0.20 mm
*Z* = 8	

### Data collection

**Table:**

Bruker APEXII CCD diffractometer	11281 independent reflections
Radiation source: fine-focus sealed tube	8711 reflections with *I* > 2σ(*I*)
Graphite monochromator	*R*_int_ = 0.052
φ and ω scans	θ_max_ = 28.0°, θ_min_ = 1.8°
Absorption correction: multi-scan (*SADABS*; Sheldrick, 2003)	*h* = −23→23
*T*_min_ = 0.862, *T*_max_ = 0.905	*k* = −18→18
52534 measured reflections	*l* = −25→25

### Refinement

**Table:**

Refinement on *F*^2^	Primary atom site location: structure-invariant direct methods
Least-squares matrix: full	Secondary atom site location: difference Fourier map
*R*[*F*^2^ > 2σ(*F*^2^)] = 0.051	Hydrogen site location: difference Fourier map
*wR*(*F*^2^) = 0.145	H-atom parameters constrained
*S* = 1.00	*w* = 1/[σ^2^(*F*_o_^2^) + (0.0795*P*)^2^ + 4*P*] where *P* = (*F*_o_^2^ + 2*F*_c_^2^)/3
11281 reflections	(Δ/σ)_max_ = 0.001
561 parameters	Δρ_max_ = 1.38 e Å^−^^3^
0 restraints	Δρ_min_ = −1.05 e Å^−^^3^

### Special details

**Table:**

Geometry. All e.s.d.'s (except the e.s.d. in the dihedral angle between two l.s. planes) are estimated using the full covariance matrix. The cell e.s.d.'s are taken into account individually in the estimation of e.s.d.'s in distances, angles and torsion angles; correlations between e.s.d.'s in cell parameters are only used when they are defined by crystal symmetry. An approximate (isotropic) treatment of cell e.s.d.'s is used for estimating e.s.d.'s involving l.s. planes.
Refinement. Refinement of *F*^2^ against ALL reflections. The weighted *R*-factor *wR* and goodness of fit *S* are based on *F*^2^, conventional *R*-factors *R* are based on *F*, with *F* set to zero for negative *F*^2^. The threshold expression of *F*^2^ > σ(*F*^2^) is used only for calculating *R*-factors(gt) *etc*. and is not relevant to the choice of reflections for refinement. *R*-factors based on *F*^2^ are statistically about twice as large as those based on *F*, and *R*- factors based on ALL data will be even larger.

### Fractional atomic coordinates and isotropic or equivalent isotropic displacement parameters (Å^2^)

**Table:**

	*x*	*y*	*z*	*U*_iso_*/*U*_eq_	
S1	0.40501 (3)	1.09117 (4)	0.46981 (3)	0.01438 (12)	
C1	0.39732 (11)	0.83090 (15)	0.37434 (11)	0.0123 (4)	
H1	0.4035	0.8478	0.3246	0.015*	
C2	0.44422 (12)	0.74267 (15)	0.39512 (12)	0.0140 (4)	
C3	0.48141 (12)	0.72432 (16)	0.46368 (12)	0.0168 (4)	
H3	0.4787	0.7700	0.5002	0.020*	
C4	0.52264 (14)	0.63971 (18)	0.47951 (14)	0.0228 (5)	
H4	0.5486	0.6284	0.5263	0.027*	
C5	0.52547 (14)	0.57230 (17)	0.42640 (14)	0.0240 (5)	
H5	0.5533	0.5145	0.4372	0.029*	
C6	0.48805 (13)	0.58815 (17)	0.35750 (14)	0.0211 (5)	
H6	0.4903	0.5416	0.3214	0.025*	
C7	0.44703 (12)	0.67319 (16)	0.34181 (12)	0.0161 (4)	
O8	0.40804 (9)	0.69639 (12)	0.27653 (8)	0.0191 (3)	
C9	0.40705 (13)	0.62893 (17)	0.21950 (12)	0.0185 (5)	
H9A	0.3817	0.5687	0.2304	0.022*	
H9B	0.4596	0.6135	0.2126	0.022*	
C10	0.36434 (13)	0.67444 (18)	0.15376 (12)	0.0196 (5)	
H10A	0.3837	0.7402	0.1484	0.024*	
H10B	0.3719	0.6366	0.1115	0.024*	
O11	0.28513 (9)	0.67811 (12)	0.15886 (8)	0.0179 (3)	
C12	0.24553 (14)	0.75651 (17)	0.12146 (12)	0.0202 (5)	
H12A	0.2291	0.7388	0.0710	0.024*	
H12B	0.2794	0.8131	0.1232	0.024*	
C13	0.17749 (13)	0.78028 (17)	0.15563 (12)	0.0191 (5)	
H13A	0.1454	0.8289	0.1272	0.023*	
H13B	0.1466	0.7220	0.1594	0.023*	
O14	0.20647 (9)	0.81722 (12)	0.22479 (9)	0.0196 (3)	
C15	0.15707 (12)	0.84805 (16)	0.26797 (12)	0.0159 (4)	
C16	0.07835 (13)	0.83922 (17)	0.25266 (13)	0.0196 (5)	
H16	0.0549	0.8126	0.2087	0.024*	
C17	0.03439 (13)	0.86984 (17)	0.30238 (14)	0.0213 (5)	
H17	−0.0193	0.8631	0.2923	0.026*	
C18	0.06749 (13)	0.91003 (16)	0.36636 (13)	0.0199 (5)	
H18	0.0369	0.9297	0.4003	0.024*	
C19	0.14625 (13)	0.92140 (16)	0.38051 (12)	0.0161 (4)	
H19	0.1691	0.9504	0.4238	0.019*	
C20	0.19172 (12)	0.89076 (15)	0.33195 (11)	0.0139 (4)	
C21	0.27704 (12)	0.89994 (15)	0.34368 (11)	0.0121 (4)	
H21	0.2925	0.9099	0.2960	0.014*	
N22	0.30377 (10)	0.98427 (13)	0.38774 (10)	0.0135 (4)	
H22N	0.2765	1.0389	0.3853	0.016*	
C23	0.37559 (12)	0.99051 (15)	0.42276 (11)	0.0123 (4)	
N24	0.42203 (10)	0.91516 (13)	0.41981 (10)	0.0134 (4)	
H24N	0.4688	0.9169	0.4458	0.016*	
N25	0.31621 (10)	0.81396 (13)	0.37458 (9)	0.0117 (3)	
C26	0.30034 (12)	0.79035 (16)	0.44608 (11)	0.0153 (4)	
H26A	0.2453	0.7853	0.4448	0.023*	
H26B	0.3207	0.8411	0.4794	0.023*	
H26C	0.3244	0.7290	0.4616	0.023*	
S2	0.59769 (3)	0.40566 (4)	0.02497 (3)	0.01375 (12)	
C27	0.59706 (11)	0.65460 (15)	0.13409 (11)	0.0124 (4)	
H27	0.5841	0.6334	0.1808	0.015*	
C28	0.55282 (12)	0.74494 (15)	0.11245 (13)	0.0157 (4)	
C29	0.52331 (13)	0.76760 (17)	0.04246 (14)	0.0212 (5)	
H29	0.5288	0.7232	0.0058	0.025*	
C30	0.48566 (15)	0.85456 (19)	0.02499 (16)	0.0306 (6)	
H30	0.4660	0.8692	−0.0231	0.037*	
C31	0.47739 (16)	0.91880 (19)	0.07820 (17)	0.0341 (7)	
H31	0.4516	0.9778	0.0665	0.041*	
C32	0.50645 (14)	0.89852 (18)	0.14926 (16)	0.0286 (6)	
H32	0.5003	0.9432	0.1856	0.034*	
C33	0.54468 (13)	0.81189 (17)	0.16622 (13)	0.0197 (5)	
O34	0.57591 (10)	0.78517 (12)	0.23351 (9)	0.0225 (4)	
C35	0.58146 (14)	0.85641 (18)	0.28819 (14)	0.0245 (5)	
H35A	0.5302	0.8747	0.2971	0.029*	
H35B	0.6069	0.9146	0.2736	0.029*	
C36	0.62663 (15)	0.81447 (19)	0.35413 (14)	0.0268 (6)	
H36A	0.6216	0.8554	0.3956	0.032*	
H36B	0.6072	0.7496	0.3628	0.032*	
O37	0.70496 (10)	0.80853 (13)	0.34550 (9)	0.0231 (4)	
C38	0.74684 (16)	0.73886 (19)	0.39038 (13)	0.0272 (6)	
H38A	0.7145	0.6822	0.3953	0.033*	
H38B	0.7636	0.7664	0.4382	0.033*	
C39	0.81451 (15)	0.70940 (18)	0.35800 (13)	0.0256 (5)	
H39A	0.8420	0.7668	0.3449	0.031*	
H39B	0.8497	0.6705	0.3922	0.031*	
O40	0.78678 (10)	0.65387 (12)	0.29591 (9)	0.0222 (4)	
C41	0.83531 (13)	0.63239 (16)	0.24964 (13)	0.0188 (5)	
C42	0.91393 (14)	0.64661 (18)	0.26350 (15)	0.0267 (6)	
H42	0.9373	0.6719	0.3080	0.032*	
C43	0.95744 (14)	0.62359 (19)	0.21210 (17)	0.0301 (6)	
H43	1.0107	0.6344	0.2215	0.036*	
C44	0.92544 (14)	0.58514 (18)	0.14725 (15)	0.0259 (6)	
H44	0.9562	0.5701	0.1124	0.031*	
C45	0.84693 (13)	0.56877 (16)	0.13378 (13)	0.0191 (5)	
H45	0.8244	0.5412	0.0898	0.023*	
C46	0.80152 (12)	0.59253 (16)	0.18437 (12)	0.0151 (4)	
C47	0.71614 (12)	0.58252 (15)	0.17053 (11)	0.0125 (4)	
H47	0.6992	0.5673	0.2168	0.015*	
N48	0.69185 (10)	0.50260 (13)	0.12106 (10)	0.0137 (4)	
H48N	0.7213	0.4497	0.1260	0.016*	
C49	0.62317 (12)	0.50040 (15)	0.07968 (11)	0.0119 (4)	
N50	0.57641 (10)	0.57561 (13)	0.08247 (10)	0.0134 (4)	
H50N	0.5309	0.5738	0.0542	0.016*	
N51	0.67883 (10)	0.67119 (13)	0.14366 (9)	0.0120 (3)	
C52	0.70353 (12)	0.70762 (16)	0.07863 (11)	0.0153 (4)	
H52A	0.7585	0.7190	0.0877	0.023*	
H52B	0.6771	0.7680	0.0644	0.023*	
H52C	0.6915	0.6602	0.0405	0.023*	
Cl1	0.27961 (4)	0.53821 (5)	0.38281 (5)	0.0428 (2)	
Cl2	0.14136 (4)	0.64065 (5)	0.40015 (4)	0.03496 (17)	
Cl3	0.16124 (4)	0.56818 (5)	0.26278 (4)	0.03620 (17)	
C53	0.20805 (15)	0.61681 (19)	0.34302 (14)	0.0251 (5)	
H53	0.2322	0.6788	0.3324	0.030*	
Cl4	0.88331 (5)	0.85664 (6)	0.13270 (5)	0.0476 (2)	
Cl5	0.75321 (6)	0.96817 (6)	0.06923 (4)	0.0536 (2)	
Cl6	0.75812 (5)	0.88555 (5)	0.21014 (4)	0.03796 (18)	
C54	0.8111 (2)	0.9377 (2)	0.14904 (15)	0.0376 (7)	
H54	0.8358	0.9976	0.1707	0.045*	

### Atomic displacement parameters (Å^2^)

**Table:**

	*U*^11^	*U*^22^	*U*^33^	*U*^12^	*U*^13^	*U*^23^
S1	0.0143 (2)	0.0117 (2)	0.0157 (3)	−0.00020 (19)	−0.00189 (19)	−0.00450 (19)
C1	0.0114 (9)	0.0128 (10)	0.0124 (10)	−0.0010 (8)	0.0013 (8)	−0.0047 (8)
C2	0.0109 (9)	0.0134 (10)	0.0175 (10)	−0.0011 (8)	0.0017 (8)	−0.0029 (8)
C3	0.0160 (10)	0.0150 (10)	0.0186 (11)	−0.0028 (8)	0.0002 (8)	−0.0024 (8)
C4	0.0211 (11)	0.0199 (12)	0.0247 (12)	−0.0003 (9)	−0.0044 (9)	0.0019 (9)
C5	0.0211 (12)	0.0141 (11)	0.0361 (14)	0.0041 (9)	0.0021 (10)	0.0009 (10)
C6	0.0196 (11)	0.0157 (11)	0.0273 (13)	0.0029 (9)	0.0017 (9)	−0.0050 (9)
C7	0.0129 (10)	0.0152 (10)	0.0201 (11)	−0.0004 (8)	0.0022 (8)	−0.0031 (8)
O8	0.0214 (8)	0.0183 (8)	0.0162 (8)	0.0037 (6)	−0.0013 (6)	−0.0087 (6)
C9	0.0180 (11)	0.0196 (11)	0.0184 (11)	−0.0006 (9)	0.0045 (9)	−0.0096 (9)
C10	0.0188 (11)	0.0243 (12)	0.0171 (11)	−0.0029 (9)	0.0068 (9)	−0.0061 (9)
O11	0.0164 (8)	0.0196 (8)	0.0182 (8)	−0.0022 (6)	0.0041 (6)	−0.0017 (6)
C12	0.0251 (12)	0.0216 (12)	0.0136 (11)	−0.0026 (9)	0.0020 (9)	−0.0023 (9)
C13	0.0187 (11)	0.0212 (11)	0.0149 (11)	−0.0016 (9)	−0.0047 (9)	−0.0059 (9)
O14	0.0135 (7)	0.0263 (9)	0.0178 (8)	−0.0010 (7)	−0.0007 (6)	−0.0107 (7)
C15	0.0140 (10)	0.0141 (10)	0.0190 (11)	0.0003 (8)	0.0012 (8)	−0.0024 (8)
C16	0.0150 (11)	0.0162 (11)	0.0252 (12)	−0.0019 (9)	−0.0042 (9)	−0.0028 (9)
C17	0.0121 (10)	0.0179 (11)	0.0333 (13)	−0.0013 (9)	0.0019 (9)	0.0008 (10)
C18	0.0173 (11)	0.0157 (11)	0.0280 (13)	0.0014 (9)	0.0075 (9)	0.0030 (9)
C19	0.0175 (11)	0.0134 (10)	0.0180 (11)	0.0012 (8)	0.0044 (8)	0.0007 (8)
C20	0.0136 (10)	0.0116 (10)	0.0160 (10)	0.0004 (8)	0.0007 (8)	−0.0002 (8)
C21	0.0123 (10)	0.0123 (10)	0.0113 (9)	−0.0005 (8)	0.0010 (7)	−0.0033 (8)
N22	0.0119 (8)	0.0113 (8)	0.0161 (9)	0.0010 (7)	−0.0013 (7)	−0.0026 (7)
C23	0.0146 (10)	0.0122 (10)	0.0102 (9)	−0.0008 (8)	0.0028 (8)	−0.0009 (7)
N24	0.0104 (8)	0.0116 (8)	0.0171 (9)	−0.0004 (7)	−0.0009 (7)	−0.0046 (7)
N25	0.0097 (8)	0.0131 (8)	0.0128 (8)	−0.0015 (7)	0.0032 (7)	−0.0021 (7)
C26	0.0154 (10)	0.0164 (10)	0.0148 (10)	−0.0012 (8)	0.0043 (8)	0.0010 (8)
S2	0.0152 (2)	0.0117 (2)	0.0135 (2)	0.00074 (19)	−0.00027 (19)	−0.00370 (19)
C27	0.0112 (9)	0.0130 (10)	0.0130 (10)	−0.0016 (8)	0.0023 (8)	−0.0030 (8)
C28	0.0099 (9)	0.0111 (10)	0.0260 (12)	−0.0013 (8)	0.0024 (8)	−0.0041 (8)
C29	0.0177 (11)	0.0147 (11)	0.0282 (13)	−0.0010 (9)	−0.0051 (9)	−0.0033 (9)
C30	0.0281 (13)	0.0183 (12)	0.0397 (16)	0.0009 (10)	−0.0117 (12)	−0.0004 (11)
C31	0.0262 (14)	0.0180 (13)	0.0537 (19)	0.0066 (10)	−0.0072 (13)	−0.0032 (12)
C32	0.0216 (12)	0.0161 (12)	0.0479 (17)	0.0024 (10)	0.0053 (11)	−0.0126 (11)
C33	0.0147 (10)	0.0152 (11)	0.0291 (13)	−0.0023 (9)	0.0031 (9)	−0.0058 (9)
O34	0.0267 (9)	0.0186 (8)	0.0231 (9)	−0.0029 (7)	0.0072 (7)	−0.0111 (7)
C35	0.0234 (12)	0.0234 (12)	0.0296 (13)	−0.0075 (10)	0.0131 (10)	−0.0164 (10)
C36	0.0314 (14)	0.0287 (13)	0.0246 (13)	−0.0110 (11)	0.0172 (11)	−0.0119 (10)
O37	0.0280 (9)	0.0235 (9)	0.0193 (9)	−0.0071 (7)	0.0083 (7)	−0.0028 (7)
C38	0.0424 (15)	0.0241 (13)	0.0146 (11)	−0.0111 (11)	0.0027 (10)	−0.0033 (9)
C39	0.0319 (14)	0.0224 (12)	0.0187 (12)	−0.0057 (10)	−0.0080 (10)	−0.0051 (9)
O40	0.0229 (8)	0.0233 (9)	0.0179 (8)	−0.0044 (7)	−0.0041 (7)	−0.0079 (7)
C41	0.0169 (11)	0.0122 (10)	0.0251 (12)	−0.0002 (8)	−0.0028 (9)	0.0022 (9)
C42	0.0178 (12)	0.0196 (12)	0.0376 (15)	−0.0043 (9)	−0.0107 (10)	0.0001 (10)
C43	0.0131 (11)	0.0193 (12)	0.0562 (18)	−0.0022 (9)	−0.0001 (11)	0.0079 (12)
C44	0.0172 (11)	0.0195 (12)	0.0425 (16)	0.0035 (9)	0.0091 (11)	0.0100 (11)
C45	0.0182 (11)	0.0143 (10)	0.0254 (12)	0.0029 (9)	0.0052 (9)	0.0045 (9)
C46	0.0123 (10)	0.0133 (10)	0.0187 (11)	−0.0007 (8)	−0.0008 (8)	0.0026 (8)
C47	0.0131 (10)	0.0128 (10)	0.0112 (9)	−0.0015 (8)	0.0010 (8)	−0.0008 (7)
N48	0.0125 (8)	0.0113 (8)	0.0161 (9)	0.0017 (7)	−0.0013 (7)	−0.0030 (7)
C49	0.0138 (10)	0.0115 (10)	0.0109 (9)	−0.0008 (8)	0.0036 (8)	0.0003 (7)
N50	0.0111 (8)	0.0113 (8)	0.0170 (9)	−0.0005 (7)	−0.0003 (7)	−0.0036 (7)
N51	0.0107 (8)	0.0123 (8)	0.0132 (8)	−0.0002 (7)	0.0022 (7)	−0.0010 (7)
C52	0.0157 (10)	0.0165 (10)	0.0145 (10)	−0.0031 (8)	0.0052 (8)	0.0011 (8)
Cl1	0.0397 (4)	0.0301 (4)	0.0545 (5)	0.0058 (3)	−0.0050 (3)	−0.0007 (3)
Cl2	0.0377 (4)	0.0322 (4)	0.0395 (4)	−0.0119 (3)	0.0199 (3)	−0.0109 (3)
Cl3	0.0401 (4)	0.0346 (4)	0.0326 (4)	0.0027 (3)	0.0016 (3)	−0.0125 (3)
C53	0.0251 (12)	0.0216 (12)	0.0295 (13)	−0.0012 (10)	0.0066 (10)	−0.0038 (10)
Cl4	0.0415 (4)	0.0501 (5)	0.0533 (5)	−0.0164 (4)	0.0138 (4)	0.0021 (4)
Cl5	0.0888 (7)	0.0322 (4)	0.0354 (4)	−0.0030 (4)	−0.0036 (4)	0.0029 (3)
Cl6	0.0564 (5)	0.0309 (4)	0.0299 (4)	−0.0131 (3)	0.0171 (3)	−0.0072 (3)
C54	0.061 (2)	0.0286 (14)	0.0242 (14)	−0.0185 (14)	0.0108 (13)	−0.0036 (11)

### Geometric parameters (Å, º)

**Table:**

S1—C23	1.700 (2)	C27—H27	1.0000
C1—N25	1.467 (3)	C28—C29	1.389 (3)
C1—N24	1.482 (3)	C28—C33	1.410 (3)
C1—C2	1.503 (3)	C29—C30	1.398 (3)
C1—H1	1.0000	C29—H29	0.9500
C2—C3	1.391 (3)	C30—C31	1.377 (4)
C2—C7	1.409 (3)	C30—H30	0.9500
C3—C4	1.395 (3)	C31—C32	1.399 (4)
C3—H3	0.9500	C31—H31	0.9500
C4—C5	1.387 (4)	C32—C33	1.397 (3)
C4—H4	0.9500	C32—H32	0.9500
C5—C6	1.392 (4)	C33—O34	1.365 (3)
C5—H5	0.9500	O34—C35	1.430 (3)
C6—C7	1.398 (3)	C35—C36	1.498 (4)
C6—H6	0.9500	C35—H35A	0.9900
C7—O8	1.363 (3)	C35—H35B	0.9900
O8—C9	1.434 (3)	C36—O37	1.435 (3)
C9—C10	1.498 (3)	C36—H36A	0.9900
C9—H9A	0.9900	C36—H36B	0.9900
C9—H9B	0.9900	O37—C38	1.423 (3)
C10—O11	1.433 (3)	C38—C39	1.498 (4)
C10—H10A	0.9900	C38—H38A	0.9900
C10—H10B	0.9900	C38—H38B	0.9900
O11—C12	1.427 (3)	C39—O40	1.433 (3)
C12—C13	1.503 (3)	C39—H39A	0.9900
C12—H12A	0.9900	C39—H39B	0.9900
C12—H12B	0.9900	O40—C41	1.364 (3)
C13—O14	1.432 (3)	C41—C42	1.399 (3)
C13—H13A	0.9900	C41—C46	1.406 (3)
C13—H13B	0.9900	C42—C43	1.381 (4)
O14—C15	1.367 (3)	C42—H42	0.9500
C15—C16	1.393 (3)	C43—C44	1.383 (4)
C15—C20	1.407 (3)	C43—H43	0.9500
C16—C17	1.390 (3)	C44—C45	1.401 (3)
C16—H16	0.9500	C44—H44	0.9500
C17—C18	1.385 (4)	C45—C46	1.395 (3)
C17—H17	0.9500	C45—H45	0.9500
C18—C19	1.396 (3)	C46—C47	1.510 (3)
C18—H18	0.9500	C47—N51	1.456 (3)
C19—C20	1.392 (3)	C47—N48	1.477 (3)
C19—H19	0.9500	C47—H47	1.0000
C20—C21	1.508 (3)	N48—C49	1.347 (3)
C21—N25	1.461 (3)	N48—H48N	0.9000
C21—N22	1.477 (3)	C49—N50	1.345 (3)
C21—H21	1.0000	N50—H50N	0.9000
N22—C23	1.349 (3)	N51—C52	1.470 (3)
N22—H22N	0.9000	C52—H52A	0.9800
C23—N24	1.343 (3)	C52—H52B	0.9800
N24—H24N	0.9000	C52—H52C	0.9800
N25—C26	1.472 (3)	Cl1—C53	1.758 (3)
C26—H26A	0.9800	Cl2—C53	1.768 (3)
C26—H26B	0.9800	Cl3—C53	1.756 (3)
C26—H26C	0.9800	C53—H53	1.0000
S2—C49	1.697 (2)	Cl4—C54	1.777 (4)
C27—N51	1.459 (3)	Cl5—C54	1.748 (3)
C27—N50	1.482 (3)	Cl6—C54	1.769 (3)
C27—C28	1.506 (3)	C54—H54	1.0000
			
N25—C1—N24	109.05 (16)	C29—C28—C33	118.7 (2)
N25—C1—C2	112.06 (17)	C29—C28—C27	123.6 (2)
N24—C1—C2	113.05 (17)	C33—C28—C27	117.6 (2)
N25—C1—H1	107.5	C28—C29—C30	121.2 (2)
N24—C1—H1	107.5	C28—C29—H29	119.4
C2—C1—H1	107.5	C30—C29—H29	119.4
C3—C2—C7	119.0 (2)	C31—C30—C29	119.4 (3)
C3—C2—C1	124.00 (19)	C31—C30—H30	120.3
C7—C2—C1	116.96 (19)	C29—C30—H30	120.3
C2—C3—C4	120.9 (2)	C30—C31—C32	121.0 (2)
C2—C3—H3	119.5	C30—C31—H31	119.5
C4—C3—H3	119.5	C32—C31—H31	119.5
C5—C4—C3	119.5 (2)	C33—C32—C31	119.3 (2)
C5—C4—H4	120.2	C33—C32—H32	120.4
C3—C4—H4	120.2	C31—C32—H32	120.4
C4—C5—C6	120.9 (2)	O34—C33—C32	124.2 (2)
C4—C5—H5	119.6	O34—C33—C28	115.4 (2)
C6—C5—H5	119.6	C32—C33—C28	120.4 (2)
C5—C6—C7	119.4 (2)	C33—O34—C35	117.75 (19)
C5—C6—H6	120.3	O34—C35—C36	107.8 (2)
C7—C6—H6	120.3	O34—C35—H35A	110.1
O8—C7—C6	124.6 (2)	C36—C35—H35A	110.1
O8—C7—C2	115.06 (19)	O34—C35—H35B	110.1
C6—C7—C2	120.3 (2)	C36—C35—H35B	110.1
C7—O8—C9	118.62 (18)	H35A—C35—H35B	108.5
O8—C9—C10	107.44 (19)	O37—C36—C35	109.2 (2)
O8—C9—H9A	110.2	O37—C36—H36A	109.8
C10—C9—H9A	110.2	C35—C36—H36A	109.8
O8—C9—H9B	110.2	O37—C36—H36B	109.8
C10—C9—H9B	110.2	C35—C36—H36B	109.8
H9A—C9—H9B	108.5	H36A—C36—H36B	108.3
O11—C10—C9	109.46 (18)	C38—O37—C36	113.41 (19)
O11—C10—H10A	109.8	O37—C38—C39	108.6 (2)
C9—C10—H10A	109.8	O37—C38—H38A	110.0
O11—C10—H10B	109.8	C39—C38—H38A	110.0
C9—C10—H10B	109.8	O37—C38—H38B	110.0
H10A—C10—H10B	108.2	C39—C38—H38B	110.0
C12—O11—C10	114.08 (18)	H38A—C38—H38B	108.4
O11—C12—C13	108.74 (19)	O40—C39—C38	107.1 (2)
O11—C12—H12A	109.9	O40—C39—H39A	110.3
C13—C12—H12A	109.9	C38—C39—H39A	110.3
O11—C12—H12B	109.9	O40—C39—H39B	110.3
C13—C12—H12B	109.9	C38—C39—H39B	110.3
H12A—C12—H12B	108.3	H39A—C39—H39B	108.6
O14—C13—C12	106.28 (18)	C41—O40—C39	118.75 (19)
O14—C13—H13A	110.5	O40—C41—C42	124.6 (2)
C12—C13—H13A	110.5	O40—C41—C46	115.6 (2)
O14—C13—H13B	110.5	C42—C41—C46	119.8 (2)
C12—C13—H13B	110.5	C43—C42—C41	119.6 (2)
H13A—C13—H13B	108.7	C43—C42—H42	120.2
C15—O14—C13	119.62 (17)	C41—C42—H42	120.2
O14—C15—C16	124.8 (2)	C42—C43—C44	121.7 (2)
O14—C15—C20	114.71 (19)	C42—C43—H43	119.2
C16—C15—C20	120.4 (2)	C44—C43—H43	119.2
C17—C16—C15	119.3 (2)	C43—C44—C45	118.9 (2)
C17—C16—H16	120.3	C43—C44—H44	120.5
C15—C16—H16	120.3	C45—C44—H44	120.5
C18—C17—C16	121.1 (2)	C46—C45—C44	120.6 (2)
C18—C17—H17	119.5	C46—C45—H45	119.7
C16—C17—H17	119.5	C44—C45—H45	119.7
C17—C18—C19	119.3 (2)	C45—C46—C41	119.4 (2)
C17—C18—H18	120.3	C45—C46—C47	122.6 (2)
C19—C18—H18	120.3	C41—C46—C47	117.9 (2)
C20—C19—C18	120.8 (2)	N51—C47—N48	110.06 (16)
C20—C19—H19	119.6	N51—C47—C46	112.00 (17)
C18—C19—H19	119.6	N48—C47—C46	111.42 (17)
C19—C20—C15	118.9 (2)	N51—C47—H47	107.7
C19—C20—C21	123.9 (2)	N48—C47—H47	107.7
C15—C20—C21	117.19 (19)	C46—C47—H47	107.7
N25—C21—N22	109.38 (16)	C49—N48—C47	122.48 (18)
N25—C21—C20	113.35 (17)	C49—N48—H48N	120.2
N22—C21—C20	112.20 (17)	C47—N48—H48N	116.3
N25—C21—H21	107.2	N50—C49—N48	118.09 (19)
N22—C21—H21	107.2	N50—C49—S2	121.49 (16)
C20—C21—H21	107.2	N48—C49—S2	120.42 (16)
C23—N22—C21	121.79 (18)	C49—N50—C27	120.93 (17)
C23—N22—H22N	115.8	C49—N50—H50N	117.5
C21—N22—H22N	121.2	C27—N50—H50N	121.5
N24—C23—N22	118.18 (19)	C47—N51—C27	107.53 (16)
N24—C23—S1	121.67 (16)	C47—N51—C52	114.02 (17)
N22—C23—S1	120.15 (16)	C27—N51—C52	112.19 (17)
C23—N24—C1	120.96 (17)	N51—C52—H52A	109.5
C23—N24—H24N	118.7	N51—C52—H52B	109.5
C1—N24—H24N	120.4	H52A—C52—H52B	109.5
C21—N25—C1	105.98 (16)	N51—C52—H52C	109.5
C21—N25—C26	113.91 (16)	H52A—C52—H52C	109.5
C1—N25—C26	112.00 (17)	H52B—C52—H52C	109.5
N25—C26—H26A	109.5	Cl3—C53—Cl1	110.29 (14)
N25—C26—H26B	109.5	Cl3—C53—Cl2	109.61 (14)
H26A—C26—H26B	109.5	Cl1—C53—Cl2	111.40 (15)
N25—C26—H26C	109.5	Cl3—C53—H53	108.5
H26A—C26—H26C	109.5	Cl1—C53—H53	108.5
H26B—C26—H26C	109.5	Cl2—C53—H53	108.5
N51—C27—N50	109.73 (16)	Cl5—C54—Cl6	111.66 (19)
N51—C27—C28	111.81 (17)	Cl5—C54—Cl4	110.18 (16)
N50—C27—C28	111.95 (17)	Cl6—C54—Cl4	109.46 (17)
N51—C27—H27	107.7	Cl5—C54—H54	108.5
N50—C27—H27	107.7	Cl6—C54—H54	108.5
C28—C27—H27	107.7	Cl4—C54—H54	108.5
			
N25—C1—C2—C3	−96.0 (2)	N51—C27—C28—C29	97.1 (2)
N24—C1—C2—C3	27.8 (3)	N50—C27—C28—C29	−26.5 (3)
N25—C1—C2—C7	81.0 (2)	N51—C27—C28—C33	−79.5 (2)
N24—C1—C2—C7	−155.28 (19)	N50—C27—C28—C33	156.93 (19)
C7—C2—C3—C4	1.6 (3)	C33—C28—C29—C30	−0.5 (3)
C1—C2—C3—C4	178.5 (2)	C27—C28—C29—C30	−177.0 (2)
C2—C3—C4—C5	−1.1 (4)	C28—C29—C30—C31	−0.3 (4)
C3—C4—C5—C6	0.3 (4)	C29—C30—C31—C32	0.4 (4)
C4—C5—C6—C7	−0.1 (4)	C30—C31—C32—C33	0.3 (4)
C5—C6—C7—O8	−179.8 (2)	C31—C32—C33—O34	179.3 (2)
C5—C6—C7—C2	0.6 (4)	C31—C32—C33—C28	−1.0 (4)
C3—C2—C7—O8	178.99 (19)	C29—C28—C33—O34	−179.2 (2)
C1—C2—C7—O8	1.9 (3)	C27—C28—C33—O34	−2.4 (3)
C3—C2—C7—C6	−1.4 (3)	C29—C28—C33—C32	1.1 (3)
C1—C2—C7—C6	−178.5 (2)	C27—C28—C33—C32	177.9 (2)
C6—C7—O8—C9	1.3 (3)	C32—C33—O34—C35	−11.2 (3)
C2—C7—O8—C9	−179.07 (19)	C28—C33—O34—C35	169.1 (2)
C7—O8—C9—C10	−176.94 (19)	C33—O34—C35—C36	−172.36 (19)
O8—C9—C10—O11	−71.7 (2)	O34—C35—C36—O37	72.6 (2)
C9—C10—O11—C12	150.54 (19)	C35—C36—O37—C38	−157.19 (19)
C10—O11—C12—C13	−154.40 (18)	C36—O37—C38—C39	156.84 (19)
O11—C12—C13—O14	67.2 (2)	O37—C38—C39—O40	−70.6 (2)
C12—C13—O14—C15	177.99 (19)	C38—C39—O40—C41	168.6 (2)
C13—O14—C15—C16	5.8 (3)	C39—O40—C41—C42	11.1 (3)
C13—O14—C15—C20	−174.6 (2)	C39—O40—C41—C46	−169.4 (2)
O14—C15—C16—C17	177.3 (2)	O40—C41—C42—C43	−179.0 (2)
C20—C15—C16—C17	−2.3 (3)	C46—C41—C42—C43	1.7 (4)
C15—C16—C17—C18	0.9 (4)	C41—C42—C43—C44	−1.1 (4)
C16—C17—C18—C19	1.1 (4)	C42—C43—C44—C45	−0.4 (4)
C17—C18—C19—C20	−1.6 (3)	C43—C44—C45—C46	1.2 (4)
C18—C19—C20—C15	0.1 (3)	C44—C45—C46—C41	−0.6 (3)
C18—C19—C20—C21	−179.6 (2)	C44—C45—C46—C47	175.8 (2)
O14—C15—C20—C19	−177.8 (2)	O40—C41—C46—C45	179.7 (2)
C16—C15—C20—C19	1.9 (3)	C42—C41—C46—C45	−0.8 (3)
O14—C15—C20—C21	1.9 (3)	O40—C41—C46—C47	3.2 (3)
C16—C15—C20—C21	−178.4 (2)	C42—C41—C46—C47	−177.4 (2)
C19—C20—C21—N25	94.1 (2)	C45—C46—C47—N51	−92.9 (2)
C15—C20—C21—N25	−85.6 (2)	C41—C46—C47—N51	83.6 (2)
C19—C20—C21—N22	−30.4 (3)	C45—C46—C47—N48	30.9 (3)
C15—C20—C21—N22	149.90 (19)	C41—C46—C47—N48	−152.65 (19)
N25—C21—N22—C23	32.5 (3)	N51—C47—N48—C49	−29.1 (3)
C20—C21—N22—C23	159.19 (19)	C46—C47—N48—C49	−153.96 (19)
C21—N22—C23—N24	−3.5 (3)	C47—N48—C49—N50	2.0 (3)
C21—N22—C23—S1	177.20 (15)	C47—N48—C49—S2	−178.82 (15)
N22—C23—N24—C1	5.2 (3)	N48—C49—N50—C27	−5.3 (3)
S1—C23—N24—C1	−175.49 (15)	S2—C49—N50—C27	175.63 (15)
N25—C1—N24—C23	−35.7 (3)	N51—C27—N50—C49	35.3 (3)
C2—C1—N24—C23	−161.11 (19)	C28—C27—N50—C49	160.02 (19)
N22—C21—N25—C1	−60.5 (2)	N48—C47—N51—C27	57.0 (2)
C20—C21—N25—C1	173.45 (17)	C46—C47—N51—C27	−178.42 (17)
N22—C21—N25—C26	63.1 (2)	N48—C47—N51—C52	−68.0 (2)
C20—C21—N25—C26	−63.0 (2)	C46—C47—N51—C52	56.5 (2)
N24—C1—N25—C21	62.2 (2)	N50—C27—N51—C47	−60.2 (2)
C2—C1—N25—C21	−171.87 (17)	C28—C27—N51—C47	174.96 (17)
N24—C1—N25—C26	−62.6 (2)	N50—C27—N51—C52	66.0 (2)
C2—C1—N25—C26	63.3 (2)	C28—C27—N51—C52	−58.9 (2)

### Hydrogen-bond geometry (Å, º)

**Table:**

*D*—H···*A*	*D*—H	H···*A*	*D*···*A*	*D*—H···*A*
N22—H22*N*···O11^i^	0.90	2.32	3.183 (2)	161
N24—H24*N*···S1^ii^	0.90	2.55	3.445 (2)	173
N48—H48*N*···O37^iii^	0.90	2.38	3.273 (3)	172
N50—H50*N*···S2^iv^	0.90	2.55	3.445 (2)	172
C10—H10*B*···S2^iv^	0.99	2.80	3.747 (2)	160
C21—H21···Cl3^i^	1.00	2.66	3.395 (2)	130
C26—H26*A*···Cl2	0.98	2.78	3.514 (2)	133
C36—H36*A*···S1^ii^	0.99	2.78	3.729 (3)	160
C43—H43···Cl3^v^	0.95	2.83	3.690 (3)	151
C53—H53···N25	1.00	2.46	3.353 (3)	149

Symmetry codes: (i) −*x*+1/2, *y*+1/2, −*z*+1/2; (ii) −*x*+1, −*y*+2, −*z*+1; (iii) −*x*+3/2, *y*−1/2, −*z*+1/2; (iv) −*x*+1, −*y*+1, −*z*; (v) *x*+1, *y*, *z*.
